# Evaluating Knowledge, Practices, and Barriers of Paediatric Pain Management among Nurses in a Tertiary Health Facility in the Northern Region of Ghana: A Descriptive Cross-Sectional Study

**DOI:** 10.1155/2020/8846599

**Published:** 2020-11-30

**Authors:** Abubakari Wuni, Solomon Mohammed Salia, Mudasir Mohammed Ibrahim, Iman Iddriss, Brenda Abena Nyarko, Samaku Nabila Seini, Imoro Tonsagri, Jauharatu Mohammed

**Affiliations:** ^1^Nurses and Midwives Training College, P.O. Box 565, Tamale, Ghana; ^2^Department of Nursing, School of Nursing and Midwifery, University of Health and Allied Sciences, Ho, Ghana; ^3^Xiangya Nursing School, Central South University, Yuelu District, Changsha, Hunan, China; ^4^Tamale Teaching Hospital, Tamale, Ghana

## Abstract

**Background:**

Pain is a major source of distress for children on admission, parents, and clinician. Hospitalized children continuously experience unrelieved pain; hence, the provision of effective pain management is an integral and important part of the nurse's role. Adequate knowledge and positive practices of nurses regarding pain management among children are key if optimal pain management is to be achieved among paediatric cases. However, there is a paucity of published data on paediatric management among nurses in the northern part of Ghana.

**Aim:**

The current study, therefore, evaluated nurse's knowledge and practices and identified the barriers to paediatric pain management in the Tamale Teaching Hospital, Ghana. *Methodology*. This was a descriptive cross-sectional facility-based study that employed a quantitative approach to data collection. A total of 180 nurses were selected conveniently from 10 selected wards of the hospital for the study. Data were collected using a questionnaire. The data were subsequently analyzed using the Statistical Package for Social Sciences version 23.0. Logistic regression analysis was done to determine the association between the dependent and independent variables of interest.

**Results:**

The findings revealed that the majority (61.1%) of all the nurses had an overall good knowledge of paediatric pain management while 57.8% demonstrated good practices of pain management. From the study, the most reported barriers to paediatric pain management by the nurses were insufficient knowledge in pain management (76.1%), inadequate paediatric pain assessment tools (73.9%), and inadequate staffing (72.2%). In further analysis, critical care nurses were 5.87 times more likely to engage in good practices of paediatric pain management than paediatric nurses (OR = 5.87 (95% CI : 1.07–32.00), *p*=0.041).

**Conclusion:**

The majority (61.1%) of all the respondents showed good knowledge of pain management and 57.8% demonstrated good pain management practices. Despite the high knowledge and practice, factors such as insufficient knowledge in pain management (76.1%), inadequate paediatric pain assessment tools (73.9%), and inadequate nurse staffing (72.2%) affect effective pain management. Paediatric pain management should be treated as a priority, and hence more efforts should be put in place to curtail the barriers that hinder its practice.

## 1. Introduction

Pain is a major source of distress for children on admission, parents, and clinicians [1]. Hospitalized children continuously experience unrelieved pain; hence, the provision of effective pain management is an integral and important part of the nurse's role [2]. The International Association for the Study of Pain (2011) defines pain as an unpleasant sensory and emotional experience from actual or potential tissue damage or described in terms of such damage. The effects of untreated or poorly managed children's pain cannot be overemphasized. Aside from the short-term biopsychosocial-developmental effects it has on the child, it can also lead to impaired pain sensitivity and long-lasting pain which is expensive to treat [3].

Clinical nurses play a vital role in managing children's pain and their accurate assessment and management of pain are necessary for positive patient outcomes [4].

However, despite the existence of protocols for effective pain control, paediatric pain is still high in children and the use of analgesics is usually inadequate [5–7].

Unrelieved pain in children may impose several unwanted physiological and psychosocial outcomes that can affect the child immediately and later in life, for instance, through increased sensitivity to later pain events [8].

Globally, studies revealed high rates of pain in hospitalized children who did not receive adequate pain management despite the increase of available treatments [9]. This seems to be the effect of inadequate knowledge of health professionals on the assessment and management of pain, as well as the presence of prejudices and barriers surrounding pain-relieving drugs [10]. Nurses per their training are often expected to have a good knowledge of pain management. However, studies showed that some nurses working with children have poor knowledge and practices with regard to paediatric pain management [2, 11, 12]. Studies by Aziato and Adejumo [13] revealed that nurses' inadequate pain management knowledge might have resulted from numerous causes such as curriculum gaps during training, inadequate clinical supervision, and lack of funding for organizing regular workshops.

Nonetheless, several other hindrances prevent nurses from optimally managing children's pain. A cross-sectional study of nurses conducted in the United States revealed deficient medication orders by physicians, inappropriate timing of premedication, low prioritization of pain management, and delays in analgesic availability as barriers to pain management [14]. Also, another study by Twycross and Collins [15] in the United Kingdom revealed a shortage of staff, a huge workload, inadequate knowledge, and shortages of pain medication as the nursing-related barriers to optimal pain care in children. Findings from Anim-Boamah [16] and Kusi Amponsah et al. [3] in the coastal part of Ghana showed that nurses had limited knowledge about pain assessment scales and pharmacological approaches for managing invasive procedural pain. The study also identified issues of communication difficulties in assessing and many other barriers to paediatric pain management.

Several studies have assessed the level of knowledge, practices, and barriers to paediatric pain management among nurses; however, there are limited studies conducted in developing countries and Ghana is no exception. Though few studies exist on paediatric pain management in the southern part of Ghana [3, 13, 16], the same cannot be said about the northern part of Ghana. There is therefore the need to conduct a study on paediatric pain management in this part of the country. Therefore, this study sought to evaluate the level of knowledge, practices, and barriers to paediatric pain management among nurses in Tamale Teaching Hospital (TTH).

## 2. Materials and Methods

### 2.1. Study Design

The study employed a descriptive cross-sectional design to assess the level of knowledge, pain management practices, and barriers to pain management among nurses at the TTH.

### 2.2. Setting

The study was carried out at the TTH which is located in the Tamale metropolis. The hospital is the largest health facility (800-bed capacity) in the northern region and serves as the only tertiary referral facility for Savana, North Eastern, Upper West, and Upper East regions, and neighboring Burkina Faso. The Tamale Metropolitan Assembly was established by a legislative instrument (LI 2068) and was subsequently elevated from Municipal Assembly into a Metropolis in 2004 with Tamale as the capital.

The hospital offers a wide range of general and specialist services. Data were collected from 10 selected wards of the hospital because these wards in one way or the other take care of children's conditions. These units were classified into paediatric units and nonpaediatric units. The paediatric units consisted of the main children's ward, the children's emergency ward, and the Neonatal Intensive Care Unit (NICU). The nonpaediatric wards included wards that nursed both adults and children and included general surgical ward, ENT ward, neurosurgical ward, plastic surgery, intensive care unit, orthopedic ward, and the main theatre recovery.

### 2.3. Study Population

The study targeted nurses working in the wards classified as paediatric and nonpaediatric units. 326 were actively engaged in nursing practice at the time of the study.

### 2.4. Inclusion and Exclusion Criteria

The study included all cadre of nurses who render clinical nursing care to children in the selected wards and who also consented to the study. The study on the other hand excluded student nurses and nurses undertaking national service as these nurses may be deemed inexperienced regarding paediatric pain management. Also, the study excluded all nurses who were on either study leave or annual leave.

### 2.5. Sampling Method and Sample Size Determination

The sample required for the study was calculated using Yammane's [17] formula for calculating sample size based on the known population. A total sample size of 180 was determined. A convenient sampling strategy was employed in selecting the nurse to participate in the study.

### 2.6. Data Collection Tool

A structured questionnaire was used for data collection regarding nurses' knowledge, practices, and barriers to pain management. The questionnaire was made up of only closed-ended questions. The tool was carefully designed, taking note of pertinent literature available and the study objectives. The tool was structured into sections: section A: demographic characteristics of nurses; section B: knowledge on paediatric pain management with 11 items; section C: pain management practices made up of 12 items; section D: barriers to paediatric pain management with 9 items. The questionnaires were serially numbered to allow for easy entry into the dataset and analysis.

### 2.7. Data Collection Procedure

Collection of data commenced after gaining approval from the Research and Development Unit of Tamale Teaching Hospital. The data collection started in October 2019 and ended in December 2019. The questionnaires were administered to nurses in the respective wards after informed consent was obtained both verbally and written. The completed questionnaires were checked for completeness and consistency of the responses and kept in a sealed envelope. Each questionnaire was coded with an identification number before giving it to the respondents for easy identification. The questionnaires were self-administered individually and clarification was however given to nurses who needed further understanding of the questions.

### 2.8. Validity and Reliability

To ensure content and face validity, the questionnaire went through peer review among 8 experts in paediatrics consisting of nurses, lecturers, and doctors. Again, the questionnaire was pretested at Tamale West Hospital among 10 nurses using the same inclusion and exclusion criteria to measure accuracy and consistency. The pretesting did not detect any ambiguities in the questions so the original form of the questionnaire was maintained.

### 2.9. Data Analysis

The data collected were subsequently entered into Microsoft Excel for cleansing and then exported to Statistical Package for Social Sciences (SPSS version 23.0.). The data were presented in the form of tables and bar charts. Numerical data were presented in frequencies and percentages. In the classification of knowledge and practices, the mean was used as a reference point. Those nurses who scored below the knowledge mean score of 5.12 were classified as having poor knowledge while those who scored above were classified as having good knowledge of pain management. Again, the nurses who scored below the practice mean score of 10.28 were classified as having poor management practice, and nurses who scored above were classified as having a good practice.

Chi-square analysis was performed to ascertain the association between knowledge, pain management practices, and sociodemographic characteristics, and other factors and *p* values of lesser than 0.05 were considered as statistically significant. Logistic regression analysis was also done to determine the relationship between independent and dependent factors at a confidence interval of 95%.

### 2.10. Ethical Considerations

Ethical approval was attained from the Tamale Teaching Hospital Research and Development unit with an official reference number: TTH/R&D/SR/126. Additional permission was obtained from the ward managers of the selected wards, and written informed consent was obtained from the nurses before administering the questionnaire to them. The respondents were properly informed and guaranteed confidentiality and anonymity throughout the study and informed that the data collection process will be done on an individual basis to ensure their privacy.

## 3. Results

### 3.1. Sociodemographic Characteristics of Respondents

Table 1 illustrates the sociodemographic descriptions of the nurses. Of the 180 nurses who took part in the study, a little above 50% (52.8%) were males and 46.1% were aged 26–30 years. The majority (82.2%) were general nurse category while only 6.1% were paediatric nurses. Less than 50% (44.4%) were within the staff nurse category while the majority (64.4%) were diploma holders.

### 3.2. Knowledge of Paediatric Pain Management

Only 15.6% of the nurses knew that children may sleep despite severe pain, and only 40.6% knew that young infants less than 6 months could tolerate opioids for pain relief. A majority (65.0%) of participants indicated wrongly that vital signs are always reliable indicators of the intensity of a child's pain while 61.7% indicated that the intravenous route is the recommended route for administering opioid analgesics in children. Furthermore, 71.7% indicated wrongly that behavioral observation scales are the most reliable measures of assessing pain and sedation in intubated/ventilated children. Nurses' knowledge of paediatric pain management is illustrated in Table 2.

### 3.3. Summary of Nurses' Knowledge on Paediatric Pain Management

**Figure 1:**
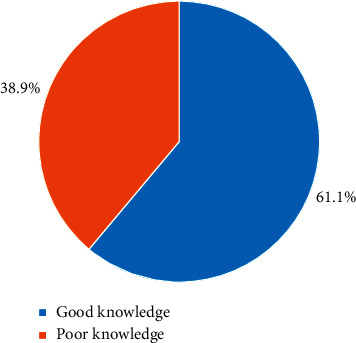
Overall nurses' knowledge on paediatric pain management.

In this study, knowledge was classified into good and poor. The study revealed that the majority (61.1%) demonstrated good knowledge while 38.9% had poor knowledge with regard to paediatric pain management as shown in Figure 1.

### 3.4. Summary of Nurses' Knowledge of Paediatric Pain Management with regard to Ward of Practice or Work

**Figure 2:**
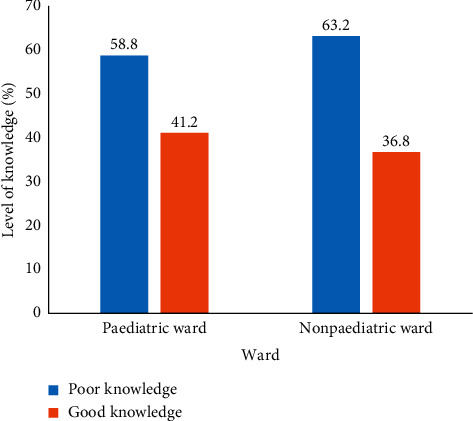
Overall level of knowledge on paediatric pain management according to ward type.

The study further revealed that nurses who worked in the paediatric wards had a higher knowledge score (41.2%) than those who worked in nonpaediatric wards (36.8%). This is illustrated in Figure 2.

### 3.5. Association between Knowledge and Sociodemographic Characteristics

Table 3 reveals that there was no significant association between knowledge and sociodemographic characteristics of the respondents.

### 3.6. Paediatric Pain Management Practices among Nurses

Most of the respondents (86.1%) did not use paediatric procedural sedation before carrying out very painful procedures in the hospital. The majority (91.1%) also indicated that they did not use pain medications during painful procedures while 90.0% observed the side effects of pain medications after they had given it to children. In the assessment of the child's pain, 77.8% used a self-reporting pain scale while 78.3% similarly used a behavioral pain scale. The majority (88.9%) used several techniques to distract children from pain. Most of the respondents (91.7%) also reassessed children's pain after pain medication was given to evaluate the effectiveness of pain medication. Most (91.7%) of the respondents reported that they talked to children in a soft voice to comfort them when they were in pain. Again, the majority of the respondents (78.9%) asked older children to support the painful areas when moving or coughing. This is shown in Table 4.

**Figure 3:**
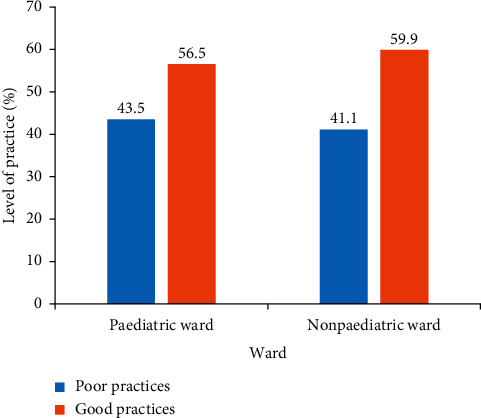
Overall level of practices on paediatric pain management according to ward type. Source: field data (2019).

With regard to the distribution of pain management practices among nurses in paediatric and nonpaediatric units, the study showed that 56.5% of respondents who worked in the paediatric wards had practiced good paediatric pain management whereas 59.9% of those who worked in nonpaediatric wards had good practices on paediatric pain management. This is shown in Figure 3.

### 3.7. Summary of Nurses Paediatric Pain Management Practices

**Figure 4:**
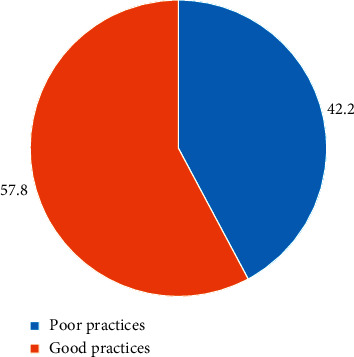
Overall level of practices on paediatric pain management. Source: field data (2019).

The study again revealed that about 57.8% and 42. 2 % of the nurses in this current study engaged in good and poor paediatric pain management practices, respectively, as shown in Figure 4.

### 3.8. Nurses Paediatric Pain Management Practices according to the Units They Work in

With regard to the distribution of pain management practices among nurses in paediatric and nonpaediatric units, the study showed that nurses who worked in nonpaediatric wards had a higher practice score (59.9%) than nurses who worked in the paediatric wards (59.9%) as shown in Figure 3.

### 3.9. Factors Associated with Nurses Paediatric Pain Management Practices

Logistic regression analysis revealed that critical care nurses were about 5.87 times more likely to engage in good practices compared to paediatric nurses regarding pain management (OR = 5.87 (95% CI : 1.07–32.00), *p*=0.041). Also, bachelor's degree and diploma holders were 10 and 8 times, respectively, more likely to engage in good practices compared to master's degree holders (OR = 10.08 (95% CI : 2.01–50.57), *p*=0.005, and OR = 8.07 (95% CI : 1.71–38.10), *p*=0.008). This is shown in Table 5.

### 3.10. Barriers Related to Paediatric Pain Management

From Table 6, the child's uncooperativeness taking medication was the most (79.4%) reported barrier related to paediatric pain management, followed by nurses' insufficient knowledge about paediatric pain assessment and management (76.1%). The majority (73.9%) also reported inadequate paediatric pain assessment tools; 72.2% reported lack of evidence-based protocol in paediatric pain management in the facility.

## 4. Discussion

The study aimed at evaluating nurses' knowledge, practices, and barriers to paediatric pain management at the largest tertiary health institution in the northern part of Ghana. The current study was necessary as it will improve nurses' pain management practices with the ultimate aim of improving the quality of paediatric care and care outcomes in the hospital. The majority of nurses in the study did not have any formal training on paediatric pain management. This is in line with a study in Rwanda by Ufashingabire et al. [11] and Zeb et al. [18] in Pakistan where most of the nurses 59.4% and 83.3%, respectively, had no formal training on paediatric pain management. Having a large number of nurses not having formal training on pain management mostly tends to influence their practice.

In this study, the majority of the nurses demonstrated good knowledge with regard to paediatric pain management. Several studies have corroborated our findings where the majority of the nurses in their respective studies reported good knowledge in paediatric pain management [18–20] in Bangladesh, the United Kingdom, and Pakistan, respectively. Despite the overall good knowledge score in the current study, the majority of the nurses demonstrated poor knowledge with regard to pain assessment and the use of analgesics in pain management among children. This finding largely agrees with the study by Alotaibi et al. [2] in Australia which revealed that nurses recorded poor knowledge in the areas of basic pain assessment and management principles. Also, the study by Anim-Boamah [16] in the southern part of Ghana revealed that nurses demonstrated limited knowledge about pain assessment scales and pharmacological strategies for managing invasive procedural pain.

Our findings further showed that nurses working in the paediatric units had a high knowledge score (41.2%) than nurses working at the nonpaediatric (36.8%). These differences in their knowledge could be due to their frequent exposure to the management of children in the paediatric wards while in the nonpaediatric wards, the management of children is scarce until a child is admitted with a specialized condition. The difference could also be due to the presence of paediatric nurses working in the paediatric wards where a transfer of knowledge could take place among the nurses.

Furthermore, the level of practice with regards to paediatric pain management in our study was generally classified as good. This finding could be attributed to a high number of nurses having good knowledge in the area. The current finding is in line with a study by Miftah et al. [21] in Ethiopia which showed that 55.8% of nurses who were involved in the study had good practices of paediatric pain management. It is however worth noting that nurses who worked in nonpaediatric wards could manage paediatric pain than those working in paediatric wards despite their high knowledge level.

Items analysis from our study showed that the majority of the nurses who were involved in the study used a self-reporting pain scale and behavioral pain scale in their pain management practices. This is corroborated with studies by Hossain et al. [19] and Baulch [22] where most nurses who took part in the study used self-reporting pain scale and behavioral pain scale in their pain management practices.

Concerning the barriers to paediatric pain management among the nurses, the majority of the nurses reported a child's uncooperativeness during pain medication administration (79.4%) and insufficient knowledge about paediatric pain assessment and management (76.1%) by nurses as the most reported barriers related to paediatric pain management. The findings of [7, 23] in Canada and the United States of America, respectively, reported similar findings. Furthermore, 73.9% of nurses in the current study also reported inadequate paediatric pain assessment tools as barrier to paediatric pain management. The findings of Kahsay and Pitkäjärvi [24] in Finland corroborate our findings where they revealed lack of protocols for pain assessment and lack of pain assessment tools to be the most perceived barriers to paediatric pain management.

Contrary to our findings on the barriers, Katende and Mugabi [25] in Uganda identified lack of time (42%), having emergencies (18%), and not knowing the right method to use (11%) as the most occurring barriers to paediatric pain management. Findings from Kusi Amponsah et al. [3] in Ghana which also disagrees with our study further reported communication difficulties in assessing and evaluating pain management interventions with children who have nonfunctional speech, misconceptions on the experience of pain in children, and an insufficient number of nurses to manage the workload and nurses' inability to prescribe analgesics as the major barriers to paediatric pain management.

## 5. Conclusion

The current study reported an overall good knowledge and practice of nurses regarding paediatric pain management. Also, nurses who worked in paediatric wards demonstrated high knowledge while those who work in nonpaediatric wards demonstrated good practice despite the majority not receiving formal training on pain management. Though some of the nurses had good knowledge and practices, there is still the need to organize regular in-service training for both nurses in paediatric and nonpaediatric units on paediatric pain management to ensure that they are up to date with the current trend in paediatric pain management. Paediatric pain management should be treated as a priority and hence more efforts should be put in place to curtail the barriers especially those related to inadequate knowledge, human, and material resources.

## Figures and Tables

**Table 1:** Sociodemographic characteristics of respondents.

Variable	Frequency (*n* = 180)	Percent
*Sex*		
Male	95	52.8
Female	85	47.2

*Age*		
≤ 25 years	23	12.8
26–30 years	83	46.1
31–35 years	48	26.7
Above 35 years	26	14.4

*Specialty*		
Paediatric nurse	11	6.1
General nurse	148	82.2
Critical care nurse	16	8.9
Others	5	2.8

*Level of education*		
Master's degree	13	7.2
Bachelor's degree	51	28.3
Diploma	116	64.4

*Rank*		
Staff nurse	80	44.4
Senior staff nurse	36	20.0
Nursing officer	35	19.5
Senior nursing officer	20	11.1
Principal nursing officer	9	5.0

*Working years of experience*		
≤2 years	37	20.6
3–5 years	57	31.7
>10 years	39	21.7
5–10 years	47	26.1

*Unit of work*		
Paediatric ward	41	22.8
Paediatrics emergency	13	7.2
Orthopedics/trauma	13	7.2
General surgery	40	22.0
Theatre recovery	14	7.8
NICU	31	17.2
Neurosurgical ward	12	6.7
Others	16	8.9

*Training on paediatric pain management*		
Yes	54	30.0
No	126	70.0

*Frequency in caring for children*		
Never	8	4.4
Often	53	29.5
Occasionally	58	32.2
Always	61	33.9

Source: field data (2019).

**Table 2:** Knowledge of paediatric pain management among nurses.

Variable	Frequency (*n* = 180)	Percent
*Children may sleep in spite of severe pain*		
Yes	28	15.6
No	152	84.4

*Underdeveloped neurological system makes children under 2 years of age have decreased pain sensitivity and limited memory of painful experiences*		
Yes	108	60.0
No	72	40.0

*Young infants, less than 6 months of age, cannot tolerate opioids for pain relief*		
Yes	107	59.4
No	73	40.6

*Vital signs are always reliable indicators of the intensity of a child's pain*		
Yes	117	65.0
No	63	35.0

*Children who can be distracted usually do not have severe pain*		
Yes	62	34.4
No	118	65.6

*Children less than 8 years old cannot reliably report pain so nurses should rely solely on parent's assessment of the child's pain intensity*		
Yes	73	40.6
No	107	59.4

*Intravenous route is the recommended route for administering opioid analgesics in children*		
Yes	111	61.7
No	69	38.3

*Children with a background of continuous, persistent pain, the oral route is used in the administration of opioid analgesics*		
Yes	89	49.4
No	91	50.6

*Analgesics for postoperative pain should initially be given only when the child asks for the medication*		
Yes	24	13.3
No	156	86.7

*Behavioral observation scales are the most reliable measures of assessing pain and sedation in intubated/ventilated children*		
Yes	126	70.0
No	54	30.0

*Opioids should not be used during the pain evaluation period, as this could mask the ability to correctly diagnose the cause of the pain if the source of the child's pain is unknown*		
Yes	129	71.7
No	51	28.3

Source: field data (2019).

**Table 3:** Association between knowledge and sociodemographic characteristics.

Variable	Knowledge level		*χ*2	*p* value	OR (95% CI), *p* value
	Poor knowledge *n* = 110 (%)	Good knowledge *n* = 70 (%)			
*Sex*					
Male	55 (50.0)	40 (57.1)			
Female	55 (50.0)	30 (42.9)	0.88	0.349	0.75 [0.41–1.37], 0.350

*Age of respondents*					
≤ 25 years	15 (13.6)	8 (11.4)			
26–30 years	50 (45.5)	33 (47.2)			1.24 [0.47–3.25], 0.665
31–35 years	29 (26.4)	19 (27.1)			1.23 [0.44–3.46], 0.697
Above 35 years	16 (14.5)	10 (14.3)	0.20	0.977	1.17 [0.37–3.76], 0.790

*Specialty*					
Paediatric nurse	6 (5.5)	5 (7.1)			
General nurse	91 (82.7)	57 (81.5)			0.75 [0.22–2.58], 0.650
Critical care nurse	11 (10.0)	5 (7.1)			0.55 [0.11–2.67], 0.455
Others	2 (1.8)	3 (4.3)	1.54	0.673	1.80 [0.21–15.41], 0.592

*Level of education*					
Master's degree	10 (9.1)	3 (4.3)			
Bachelor's degree	29 (26.4)	22 (31.4)			2.53 [0.62–10.30], 0.195
Diploma	71 (64.5)	45 (64.3)	1.76	0.416	2.11 [0.55–8.09], 0.275

*Rank*					
Staff nurse	51 (46.4)	29 (41.4)			
Senior staff nurse	19 (17.3)	17 (24.3)			1.57 [0.71–3.49], 0.265
Nursing officer	19 (17.3)	16 (22.9)			1.48 [0.66–3.20], 0.340
Senior nursing officer	15 (13.6)	5 (7.1)			0.59 [0.19–1.78], 0.346
Principal nursing officer	6 (5.4)	3 (4.3)	3.71	0.446	0.88 [0.20–3.78], 0.863

*Years of work experience*					
≤2 years	22 (20.0)	15 (21.4)			
3–5 years	37 (33.6)	20 (28.6)			0.79 [0.34–1.86], 0.593
>10 years	23 (20.9)	16 (22.9)			1.02 [0.41–2.55], 0.966
5–10 years	28 (25.5)	19 (27.1)	0.51	0.917	0.99 [0.41–2.39], 0.991

*Unit of work*					
Paediatrics	22 (20.0)	19 (27.1)			
Paediatrics emergency	10 (9.1)	3 (4.3)			0.35 [0.08–1.45], 0.147
Orthopedics/trauma	11 (10.0)	2 (2.9)			0.21 [0.04–1.07], 0.060
General surgery	29 (26.4)	19 (27.1)			0.76 [0.33–1.76], 0.521
Theatre recovery	10 (9.1)	4 (5.7)			0.46 [0.12–1.72], 0.250
NICU	18 (16.4)	13 (18.6)			0.84 [0.33–2.14], 0.710
Other surgeries	5 (4.5)	7 (10.0)			1.62 [0.44–5.96], 0.467
Others	5 (4.5)	3 (4.3)	8.02	0.331	0.69 [0.15–3.30], 0.647

*Formal training on paediatric pain management*					
Yes	29 (26.4)	25 (35.7)			
No	81 (73.6)	45 (64.3)	1.78	0.182	0.64 [0.34–1.23], 0.183

Source: field data (2019).

**Table 4:** Paediatric pain management practices.

Variable	Frequency (*n* = 180)	Percent
*Use of policy in hospital for paediatric procedural sedation*		
Yes	25	13.9
No	155	86.1

*Use of pain medications/sedation during painful procedure*		
Yes	16	8.9
No	164	91.1

*Observation of side effects of pain medication after giving it to children*		
Yes	162	90.0
No	18	10.0

*Placing of children in comfortable positions to help relieve pain*		
Yes	172	95.6
No	8	4.4

*Use of self-reporting pain scale to assess children's pain*		
Yes	140	77.8
No	40	22.2

*Use of behavioral pain scale to assess children's pain*		
Yes	141	78.3
No	39	21.7

*Use of several techniques to distract children from pain*		
Yes	160	88.9
No	20	11.1

*Reassessing children's pain after given pain medication to evaluate the effectiveness of pain medication*		
Yes	165	91.7
No	15	8.3

*Administration of additional pain medication to relieve pain when needed*		
Yes	170	94.4
No	10	5.6

*Talking to children with a soft voice to comfort them when they are in pain*		
Yes	165	91.7
No	15	8.3

*Asking and helping children to support the painful areas when moving or coughing*		
Yes	142	78.9
No	38	21.1

Source: field data (2019).

**Table 5:** Logistic regression results showing factors associated with nurses' paediatric pain management practices.

Variable	Practices level		*χ*2	*p* value	OR (95% CI), *p* value
	Poor practices *n* = 76 (%)	Good practices *n* = 104 (%)			
*Sex*					
Male	42 (55.3)	53 (51.0)			
Female	34 (44.7)	51 (49.0)	0.33	0.568	1.19 [0.66–2.15], 0.568

*Age group*					
≤25 years	11 (14.5)	12 (11.5)			
26–30 years	28 (36.8)	55 (52.9)			1.80 [0.71–4.59], 0.218
31–35 years	24 (31.6)	24 (23.1)			0.92 [0.34–2.48], 0.864
Above 35 years	13 (17.1)	13 (12.5)	4.58	0.205	0.92 [0.29–2.82], 0.879

*Specialty*					
Paediatric nurse	8 (10.5)	3 (2.9)			
General nurse	61 (80.3)	87 (83.6)			3.80 [0.97–14.92], 0.055
Critical care nurse	5 (6.6)	11 (10.6)			5.87 [1.07–32.00], 0.041
Others	2 (2.6)	3 (2.9)	5.06	0.168	4.00 [0.43–37.12], 0.223

*Level of education*					
Master's degree	11 (14.5)	2 (1.9)			
Bachelor's degree	18 (23.7)	33 (31.7)			10.08 [2.01–50.57], 0.005
Diploma	47 (61.8)	69 (66.4)	10.72	0.005	8.07 [1.71–38.10], 0.008

*Rank*					
Staff nurse	35 (46.0)	45 (43.3)			
Senior staff nurse	12 (15.8)	24 (23.1)			1.56 [0.68–3.54], 0.292
Nursing officer	10 (13.2)	25 (24.0)			1.94 [0.83–4.58], 0.128
Senior nursing officer	12 (15.8)	8 (7.7)			0.52 [0.19–1.41], 0.197
Principal nursing officer	7 (9.2)	2 (1.9)	11.17	0.025	0.22 [0.04–21.14], 0.071

*Years of work experience*					
≤2 years	12 (15.8)	25 (24.0)			
3–5 years	30 (39.5)	27 (26.0)			0.43 [0.18–1.02], 0.057
>10 years	16 (21.0)	23 (22.1)			0.69 [0.27–1.76], 0.438
5–10 years	18 (23.7)	29 (27.9)	4.31	0.230	0.77 [0.31–1.91], 0.578

*Unit of work*					
Paediatrics	20 (26.3)	21 (20.2)			
Paediatrics emergency	4 (5.3)	9 (8.7)			2.14 [0.57–8.08], 0.260
Orthopedics/trauma	5 (6.6)	8 (7.7)			1.52 [0.43–5.45], 0.517
General surgery	22 (29.0)	26 (25.0)			1.13 [0.49–2.59], 0.781
Theatre recovery	6 (7.9)	8 (7.7)			1.27 [0.37–4.31], 0.702
NICU	13 (17.1)	18 (17.3)			1.32 [0.36–3.38], 0.564
Other surgeries	5 (6.6)	7 (6.7)			1.33 [0.36–4.89], 0.665
Others	1 (1.3)	7 (6.7)	4.66	0.702	6.67 [0.75–59.15], 0.089

*Formal training on paediatric pain management*					
Yes	22 (28.9)	32 (30.8)			
No	54 (71.1)	72 (69.2)	0.07	0.792	0.92 [0.48–1.75], 0.792

**Table 6:** Barriers related to paediatric pain management.

Variable	Frequency (*n* = 180)	Percent
*Insufficient knowledge about paediatric pain assessment tool and management by nurses*		
Yes	137	76.1
No	43	23.9

*Child's uncooperativeness taking medication*		
Yes	143	79.4
No	37	20.6

*Lack of evidence-based protocol in paediatric pain management in facility*		
Yes	130	72.2
No	50	27.8

*Low priority given to pain management by staff*		
Yes	98	54.4
No	82	45.6

*Inadequate paediatric pain assessment tools*		
Yes	133	73.9
No	47	26.1

*Staff concern about side effects of medication (other than addiction)*		
Yes	121	67.2
No	59	32.8

*Inadequate staffing and other resources*		
Yes	130	72.2
No	50	27.8

*Insufficient cooperation by doctor in relation to suggestions by the nurse on pain prescription*		
Yes	95	52.8
No	85	47.2

*Staff concern about children becoming tolerant to analgesics*		
Yes	121	67.2
No	59	32.8

Source: field data (2019).

## Data Availability

The data used for this study were collected from Tamale Teaching Hospital and are available on request from the corresponding author.
